# Using a Standing-Tree Acoustic Tool to Identify Forest Stands for the Production of Mechanically-Graded Lumber

**DOI:** 10.3390/s130303394

**Published:** 2013-03-12

**Authors:** Normand Paradis, David Auty, Peter Carter, Alexis Achim

**Affiliations:** 1 Centre de recherche sur le bois, Université Laval, 2425 rue de la Terrasse, Québec, QC G1V 0A6, Canada; E-Mails: normand.paradis.1@ulaval.ca (N.P.); auty.david.1@ulaval.ca (D.A.); 2 Fibre-Gen, Unit 5 Amuri Park, 404 Barbadoes Street, Christchurch 8013, New Zealand; E-Mail: peter.carter@fibre-gen.com

**Keywords:** acoustic sensors, forestry wood chain, wood stiffness, machine stress-rated lumber

## Abstract

This study investigates how the use of a Hitman ST300 acoustic sensor can help identify the best forest stands to be used as supply sources for the production of Machine Stress-Rated (MSR) lumber. Using two piezoelectric sensors, the ST300 measures the velocity of a mechanical wave induced in a standing tree. Measurements were made on 333 black spruce (*Picea mariana* (Mill.) BSP) trees from the North Shore region, Quebec (Canada) selected across a range of locations and along a chronosequence of elapsed time since the last fire (TSF). Logs were cut from a subsample of 39 trees, and sawn into 77 pieces of 38 mm × 89 mm cross-section before undergoing mechanical testing according to ASTM standard D-4761. A linear regression model was developed to predict the static modulus of elasticity of lumber using tree acoustic velocity and stem diameter at 1.3 m above ground level (R^2^ = 0.41). Results suggest that, at a regional level, 92% of the black spruce trees meet the requirements of MSR grade 1650Fb-1.5E, whilst 64% and 34% meet the 2100Fb-1.8E and 2400Fb-2.0E, respectively. Mature stands with a TSF < 150 years had 11 and 18% more boards in the latter two categories, respectively, and therefore represented the best supply source for MSR lumber.

## 1. Introduction

The boreal forest zone of northeastern North America is a major source of softwood lumber for both domestic and export markets [1]. However, wood processing industries in the region have been severely affected by the recent global economic downturn and the resulting fall in demand for structural wood products [1,2]. In remote areas (e.g., northern Quebec, Canada), the situation has been exacerbated by high harvesting and transport costs, which have increased the pressure to add value to the existing resource [3,4]. One way of achieving this is by diversifying output through the manufacture of value-added products such as machine stress-rated (MSR) lumber [5,6].

The black spruce (*Picea mariana* (Mill.) BSP) and balsam fir (*Abies balsamea* (L.) Mill.) dominated stands of the boreal forest [7] offer the potential to increase the production of MSR lumber for structural applications [8–11]. This is due to the typically higher mechanical properties of wood from mature, slow-grown stands [8]. However, MSR lumber production currently represents only a small percentage of the total dimension lumber output when compared to visually-graded products [9]. Moreover, the large inherent variability in stand structure, species composition and ultimately stem quality, may lead to concerns about overall pass-rates under machine grading systems [10].

For these reasons, forest managers are increasingly recognizing the importance of resource quality characterization earlier along the value chain. Ideally this information will be used to inform planning decisions so that raw material can be allocated to appropriate markets according to its predicted end-use properties [12]. As the design values of MSR lumber are derived from characteristic thresholds of lumber stiffness (modulus of elasticity, MOE) and strength (modulus of rupture, MOR), more accurate predictions of mean mechanical properties at the stand or regional scale could improve pass-rates by segregating poorer material prior to processing [13,14].

Recently, new acoustic sensors have been developed for the rapid nondestructive evaluation (NDE) of standing trees and logs. These instruments are used to estimate MOE through its relationship with stress-wave velocity or acoustic resonance [15–19]. Assessing wood stiffness prior to processing can assist decision-making along a complex supply chain characterized by divergent processes [20]. Accordingly, acoustic resonance tools for the assessment of logs are increasingly being deployed in sawmills to streamline log sorting and improve grade recovery of veneers and structural products [19]. The uptake of standing tree acoustic tools, however, has been slower, largely due to the relatively weaker relationship between stress-wave velocity and the static MOE of sawn boards [18]. However, standing tree tools are useful for providing stand-level estimates of mean wood stiffness prior to harvest, which can inform resource allocation decisions for individual forest stands.

The aim of this study was to use standing tree assessments of wood stiffness to predict structural grade outturn in black spruce trees. Specific objectives were as follows: (1) to develop a linear regression equation linking mean tree MOE with stem diameter and acoustic velocity; (2) to use logistic regression to predict the proportion of boards that meet the requirements of certain MSR grades as a function of the predicted tree-level MOE, and (3) to combine steps 1 and 2 using inventory data to predict the MSR potential of the black spruce resource at the regional scale. The effect of stand structure on MSR grade potential was also tested by sampling from stands at different post-fire successional stages.

## 2. Materials and Methods

### 2.1. Description of Acoustic Sensor

The Hitman ST300 (Fibre-gen, Christchurch, New Zealand) is a portable device designed to measure the velocity of mechanical stress-waves in standing trees [14,21]. Theoretically, acoustic velocity measurements are directly related to the dynamic modulus of elasticity and density by the one-dimensional wave equation:

(1) $$V=\sqrt{\frac{{\text{MOE}}_{D}}{\rho }}$$

where *V* is the acoustic velocity (m·s^−1^), *MOE_D_* is the dynamic modulus of elasticity (N·m^−2^) and *ρ* is the wood density (kg·m^−3^). Since the tool does not provide a measure of green density, it is usually assumed to be constant for a given species and time of year, to account for seasonal fluctuations in moisture content [17,22]. In addition, stem diameter is known to induce variation in stress-wave velocity, even if *MOE_D_* and *ρ* remain constant [16–18].

**Figure 1.**
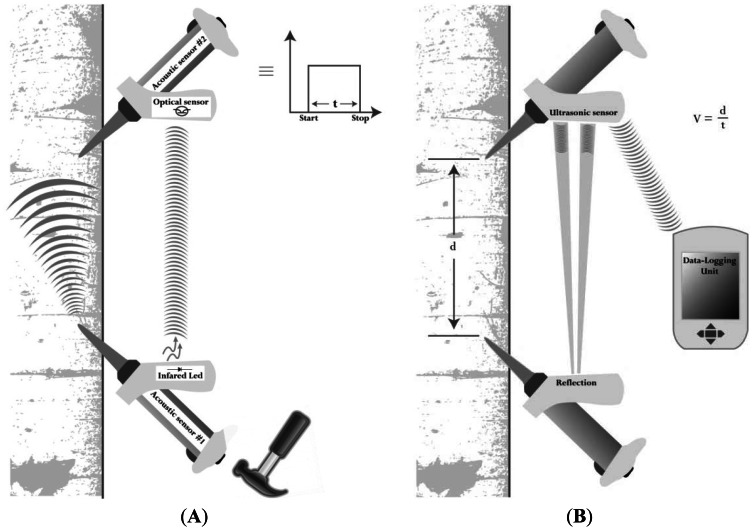
Schematic diagram of the ST300 operating principle. (**A**) The start of the mechanical wave is detected with an infrared signal (**B**) The distance traveled by the wave is measured using ultrasonic reflection between the two probes. Data are transferred wirelessly to the data logger.

The tool contains two Monitran MTN/P100 accelerometers, each attached to a probe inserted into the lower part of the stem at a 45-degree angle and aligned vertically between 50 and 120 cm apart (Figure 1).

The accelerometer in the lower probe detects the stress-wave induced by a hammer blow, while the second accelerometer records the arrival time of the stress wave. The transit time *t* of the mechanical wave between probe tips is derived from the raw elapsed time by deducting the (constant) time taken for the stress wave to travel through the metal probe tips. The distance *d* between the probes is measured using ultrasonic sensors, and after the distance from each sensor to the respective probe tip is deducted, the acoustic velocity is easily calculated as *d*/*t*. The system is similar to a timer in which the triggering signal associated with the hammer impact on the first probe is transmitted by an infrared beam to the main circuit, which itself is connected to the second piezoelectric accelerometer. The timer is stopped once the second sensor detects the vibration from the mechanical wave. Due to inherent variability between successive readings on the same tree, the tool provides the average velocity from eight successive hits. This number was carefully selected to limit the variability of the middle four out of eight hits to be typically around 2% on an average time of flight reading of 300 μs. Results can be read directly on the instrument display panel as well as being transmitted wirelessly to a data logger (Figure 1).

### 2.2. Study Area and Sampling Locations

**Figure 2.**
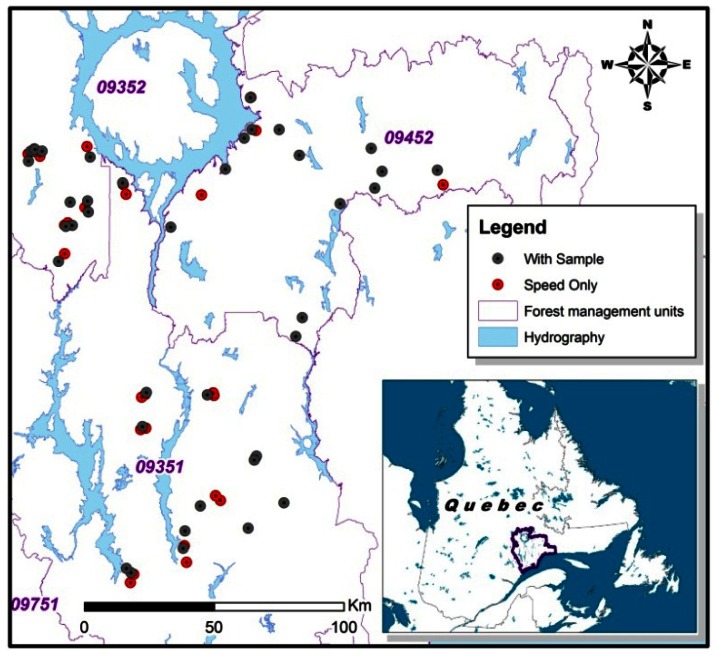
Location of sample plots in the North Shore region of Quebec, Canada.

The sample trees were located over a large area (20,000 km^2^) in the North Shore region of Quebec, Canada (Figure 2). In this part of the boreal forest, fire disturbance is the primary driver of stand structural dynamics. Immediately following a stand-initiating fire, the black spruce-dominated stands form dense cohorts of even-aged, uniform new growth. However, as the elapsed time since the last fire (TSF) increases beyond 150–200 years, stands gradually become more uneven-aged, while the proportion of balsam fir (*Abies balsamea* (L.) Mill.) increases [7,23,24]. In the North-Shore region, the average fire return interval is estimated to be over 500 years [23]. Consequently, more than 60% of stands in the region are irregular in structure, with greater variability in stem size, slower growth rates and an increasing proportion of dead stems [23,25,26]. To test the effect of stand structure on predicted grade recovery, stands were selected from four categories of TSF: 50–99, 100–149, 150–199, and >200 years. The location of sampling sites was based on a chronosequence of mean fire return intervals developed in a previous study [23].

### 2.3. Standing Tree Acoustic Measurements

Since moisture content affects density and therefore the speed of propagation of a stress-wave in wood, acoustic measurements were taken on the sample trees around 1.3 m from ground level with the probes inserted into the stem at two different depths, namely 1.5 cm and 3 cm, which conform to previously published data on sapwood and heartwood thickness [27]. To limit the potential influence of environmental effects on wood properties, such as the prevailing wind direction, acoustic readings were taken on both the south- and east-facing cardinal direction on each tree. In total, the NDE dataset consisted of 333 black spruce trees from 67 plots. A subsample of 39 trees, each collected in separate plots, was then randomly selected for destructive testing. After felling, a 1.8 m log was collected on each tree from between 1.3 and 3.1 m along the stem and transported to Laval University's Wood Research Center for processing into boards for mechanical testing.

The sampling locations along the chronosequence were selected on the basis of accessibility to the existing road network. In addition, every effort was made to sample trees from distinct recorded fire events. At each location, a random distance (50 m to 200 m from the road) and a random azimuth were chosen to determine the exact location of the plot. Variable-radius plots were established using a prism of factor 2 [28,29]. Starting from a random direction, the first five trees included in the plot were selected for acoustic velocity measurements. Tree diameter at breast height (DBH, cm—measured 1.3m from ground level) was also recorded.

### 2.4. Mechanical Tests on Sawn Pieces

The 39 harvested logs were sawn using a portable sawmill into 1.8 m-long boards with nominal dimensions of 38 mm × 89 mm in cross section, after drying and planing. The sawn pieces were stored at 20 °C and 65% RH for five months, until they reached an equilibrium moisture content of approximately 12%. Mechanical properties were assessed in accordance with ASTM D4761 [30]. The tests were performed using the following specifications: edge-wise loading, span length of 160 cm, three-point bending and span-to-depth ratio of 18. The static MOE (kN·mm^−2^) was calculated using the stress values recorded between 10% and 45% of the maximum load at failure for each piece, thus ensuring the elastic limit was not reached. Values were adjusted to a moisture content of 15% following ASTM D1990 [31], which corresponds to the requirements associated with the production of MSR lumber [32]. Prior to the bending tests, each board had been inspected and only those pieces meeting the requirements of the No. 2 & Better visual grade mechanically tested. Finally, the tree-level MOE was calculated as the arithmetic mean of the MOE measurements from all boards sawn from each tree.

### 2.5. Statistical Analysis

First, the averaged MOE data were used to develop a multivariate linear regression model describing mean tree MOE as a function of the acoustic velocity squared and tree diameter. An interaction term between these variables was also included in the model, and the root mean square error (RMSE) and mean percentage error (E%) calculated from the observed and predicted values. The explanatory variables were centered on their mean values prior to model-fitting to reduce the effects of multicollinearity and increase the interpretability of the model coefficients [33]. The parameters of this equation were then used to estimate mean stiffness values (MOE_est_, kN·mm^−2^) for each tree in the larger NDE dataset. Next, analysis of variance (ANOVA) was carried out to determine if there were significant differences in mean acoustic velocity and mean MOE_est_ between the TSF classes. Multiple comparisons were made using Tukey's honestly significant difference (HSD) tests, which identify any significant differences between groups.

Since each board was assigned an MSR grade based on its static MOE value, logistic regression was used to model the proportion of boards meeting the requirements of certain MSR classes as a function of the predicted tree-level stiffness values (MOE_est_). Logistic regression can model dichotomous outcomes as binary variables that are coded as either 1 or 0, with the proportion of 1s representing the probability (between 0 and 1) of the event of interest occurring [34]. In this case, the vector of 1s in the data represented the situation where the stiffness requirements for the grade of interest were attained. For this purpose, boards were assigned to one of three MSR grades—1650Fb-1.5E (*i.e.*, 11.4 MPa and 10.3 GPa for Fb and E, respectively), 2100Fb-1.8E (14.5 MPa–12.4 GPa) and 2400Fb-2.0E (16.6 MPa–13.8 GPa)—according to specifications defined in the SPS-2 standard produced by the National Lumber Grade Authority (NLGA) [32]. In this system, grade names refer to (1) the average MOE for the group in Mpsi (e.g., 1.5E) and (2) a MOR (psi) threshold corresponding the 95th percentile of the group divided by 2.1 (e.g., 1650 Fb).

**Figure 3.**
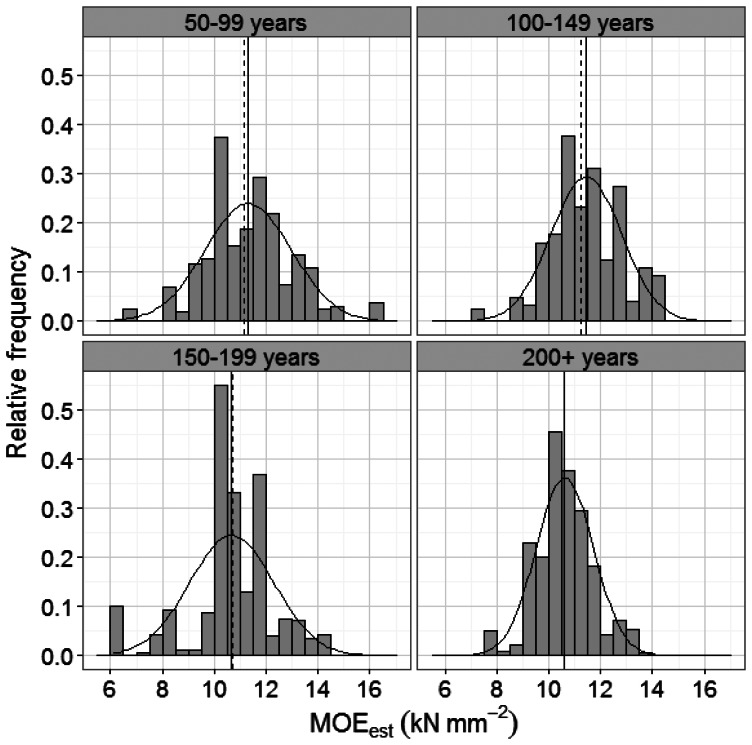
Normal distribution curves overlaid on histograms of MOE_est_ for each TSF class. The dashed line on each graph represents the median and the black line represents the mean.

Subsequently, the information from the linear and logistic regression equations was used to estimate the proportion of sawn pieces that could be expected to meet a given MSR grade for each TSF class. This was achieved in a 3-stage process. First, the measurements made in the variable-radius plot were expressed on a per hectare basis. Since the plot radius varied with the DBH of the stems, observations had to be weighted using a correction factor of 80,000/(π·DBH^2^) [24]. A visual inspection of histograms of MOE_est_ values overlaid with normal density curves confirmed that the data in each TSF class could be assumed to be normally distributed (Figure 3). This assumption allowed the mean and standard deviation of the probability density function of MOE_est_ to be calculated for each TSF class. The relative frequency distribution function of a variable *x* is given by:

(2) $$P(x)=\frac{1}{\sigma \sqrt{2\pi }}e^{-{\left(x-\mu \right)}^{2}/2\sigma^{2}}$$

where *μ* and *σ* are mean and standard deviation, respectively, of the normally distributed variable *x*. Secondly, the predicted probability of meeting the requirements of a specific MSR grade were calculated using the inverse logit link:

(3) $${\text{logit}}^{-1}(\theta_{1}+\theta_{2}\times {\text{MOE}}_{\text{est}})=\frac{e^{\left(\theta_{1}+\theta_{2}\times {\text{MOE}}_{\text{est}}\right)}}{1+e^{\left(\theta_{1}+\theta_{2}\times {\text{MOE}}_{\text{est}}\right)}}$$

where *θ*_1_ + *θ*_2_ × *MOE_est_* represents the predicted value on the link scale from the vector of logistic regression coefficients *θ*_1_ and *θ*_2_ for each MSR grade, modelled as a function of MOE_est_. Thirdly, predicted pass rates for each MSR grade in each TSF class were calculated as the integral of the product of Equations (2) and (3) as follows:

(4) $$\text{Pass rate}=\int_{{\text{MOE}}_{\text{est}}\min}^{{\text{MOE}}_{\text{est}}\max}\frac{1}{\sigma \sqrt{2\pi }}e^{-{\left({\text{MOE}}_{\text{est}}-\mu \right)}^{2}/2\sigma^{2}}\times \frac{e^{\left(\theta_{1}+\theta_{2}\times {\text{MOE}}_{\text{est}}\right)}}{1+e^{\left(\theta_{1}+\theta_{2}\times {\text{MOE}}_{\text{est}}\right)}}$$

All analyses were performed using functions contained in the R statistical programming environment (R Development Core Team, 2012).

## 3. Results and Discussion

### 3.1. Linear Regression Predicting MOE from Acoustic Velocity and Diameter

**Figure 4.**
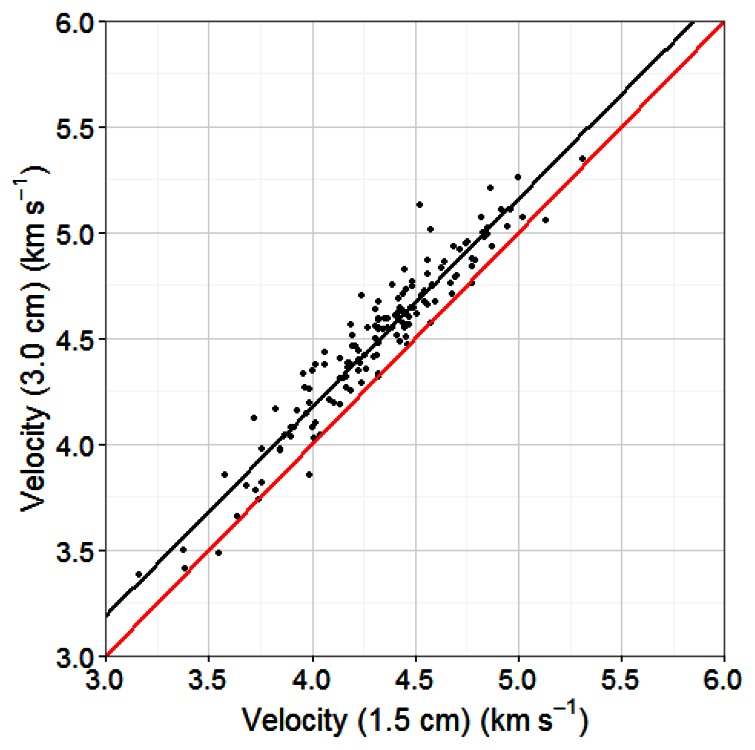
Relationship between standing tree acoustic velocity measured at horizontal depths of 1.5 and 3.0 cm in the stem (R^2^ = 0.91).

Acoustic velocities at probe depths of 1.5 and 3 cm were highly correlated (*R*^2^ = 0.91) and thus appeared to have a similar potential for MOE predictions. However, they were consistently higher at a depth of 3 cm than at 1.5 cm, with mean values of 4.39 and 4.32, respectively (Figure 4). Insertion depth could therefore be an important factor to control for in any applications of this technology at an industrial scale. For the current study, all further results use the acoustic velocities measured at a depth of 3 cm.

Mean tree static MOE was positively related to the acoustic velocity squared and negatively to tree diameter. There was also an interaction between the velocity squared and diameter terms. The equation for MOE_est_ was given by:

(5) $${\text{MOE}}_{\text{est}}=10.680+0.2362\times V^{2}-0.1252\times \text{DBH}-0.0649\times V^{2}\times \text{DBH}$$

Because the explanatory variables were centered on their mean values, the model intercept of 10.680 is the estimated MOE when both explanatory variables are set to their mean values. The coefficient of determination (*R*^2^) of the relationship between the observed and predicted MOE was 0.41, which is lower than the values of 0.65 and 0.55 previously reported by Mora *et al.* and Liu *et al.*, repsectively [17,34]. In the latter case, DBH was also used to predict MOE, but the model also included crown length, stem taper and stand density as explanatory variables. However, our results are comparable to those of Wang *et al.* and Eckard who reported *R*^2^ values of 0.44 and 0.45, respectively [35,36]. The RMSE and E% values calculated from the predicted and observed MOE values in our study were low (1.06 kN·mm^−2^ and 0.01%, respectively). The overall fit of the model was hence judged sufficient to pursue our analyses.

The interaction term in our model indicates that the effect of acoustic velocity on MOE is mediated by stem diameter. This may occur due to the anisotropic nature and heterogeneous structure of wood [37] or, more simply, to the type of measurement that is made. The theory of one-dimensional plane waves describes behaviour in an unconstrained, homogenous slender rod with a diameter-to-length ratio smaller than 0.1 [18]. In trees, wood properties vary considerably from pith to bark. For black spruce, density and MOE increase with cambial age from juvenile (less than 10–15 year) to mature wood [38]. The moisture content of wood also varies within the tree from around 40% (heartwood) to well over 100% (sapwood) [8]. All three of these properties—MOE, density and moisture content—have an influence on the propagation of mechanical waves, and may interact in complex ways in anisotropic materials such as wood.

When the wave is generated by the impact of the hammer, it first spreads through the stem at an angle of 45 degrees (dilatational wave). However, the wave front soon evolves into a quasi-plane wave propagating in the longitudinal direction of the stem [39]. Since the wave travels more slowly in wetter wood, the most advanced part of the wave front should be located just inside the drier heartwood. This zone is typically located at a radial depth of approximately 3 cm in mature black spruce [27], a fact that would explain the higher velocity readings.

### 3.2. Multiple Comparisons between TSF Classes

Values of acoustic velocity, DBH and MOE_est_ in the NDE dataset (n = 333) showed some variation according to TSF classes (Table 1). Mean acoustic velocity was significantly higher in stands with a TSF of 100–149 years than in stands from all other TSF classes, which were also not significantly different from each other. For MOE_est_, values in the two youngest TSF classes were significantly higher than in stands with a TSF greater than 200 years. The only significant differences in mean DBH were between stands in the 50–99 year TSF class and all the older classes. The results suggest that even-aged, uniform stands initiated by a forest fire may produce wood with higher structural performance than stands in which the last fire occurred more than 200 years ago. Further investigations are underway that will provide more data to elucidate the influence of TSF on the structural wood properties of this important resource, particularly considering the high proportion (>60%) of stands with a TSF greater than 200 years in our study area [25].

### 3.3. Stand-Level Assessment of MSR Lumber Grades

The parameters of the logistic regression equations for the proportion of boards meeting the requirements of each MSR grade are presented in Table 2. MOE_est_ did not significantly influence the proportion of boards meeting the design specifications of the 1650Fb-1.5E grade, although it was a significant predictor of the two higher grades.

The stand-level predictions of the percentage of the resource meeting the minimum requirements of each selected MSR grade, calculated using Equation (4), are shown in Table 3. Apart from the 1650Fb-1.5E grade predictions, which were consistently high (92%) across all TSF classes, the pass rates for each of the higher grades were lowest in stands with TSF greater than 150 years. Additionally, within each TSF class, pass rates decreased from the lowest to the highest grade. The pass rates for the 1650Fb-1.5E grade are higher than those predicted by Zhang *et al.*, who estimated that approximately 80% of sawn pieces would meet the corresponding requirements under visual grading rules (No. 2 and better [35]) for black spruce growing in natural stands [10]. However this percentage may be reduced depending on certain stand-level characteristics, such as the proportion of decayed or windthrown stems [3,40,41]. In addition, sawing defects that can cause downgrades, such as wane, were avoided in our study.

**Figure 5.**
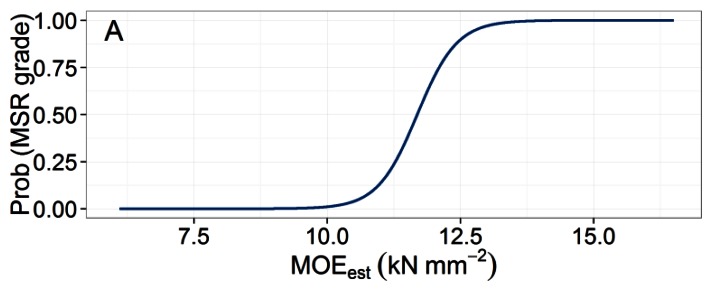

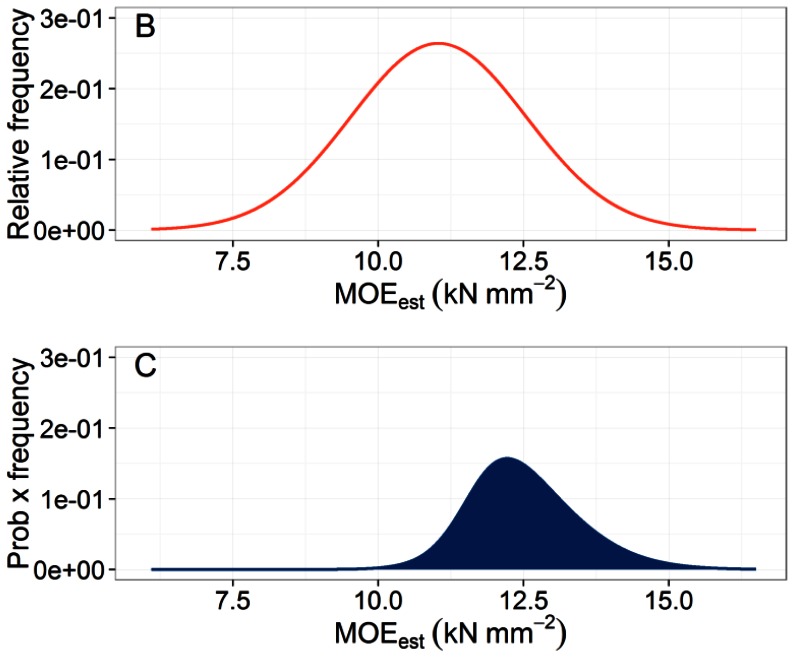
(**A**) Proportion of boards meeting the requirements of the 2400Fb-2.0E MSR grade for a given value of MOE_est_ assigned to a tree using the ST-300. (**B**) Relative frequency distribution of MOE_est_ values. (**C**) Probability of meeting grade requirements multiplied by normal relative frequency distribution. The integral of the resulting curve provides a stand-level pass rate prediction for the specified MSR grade.

**Figure 6.**
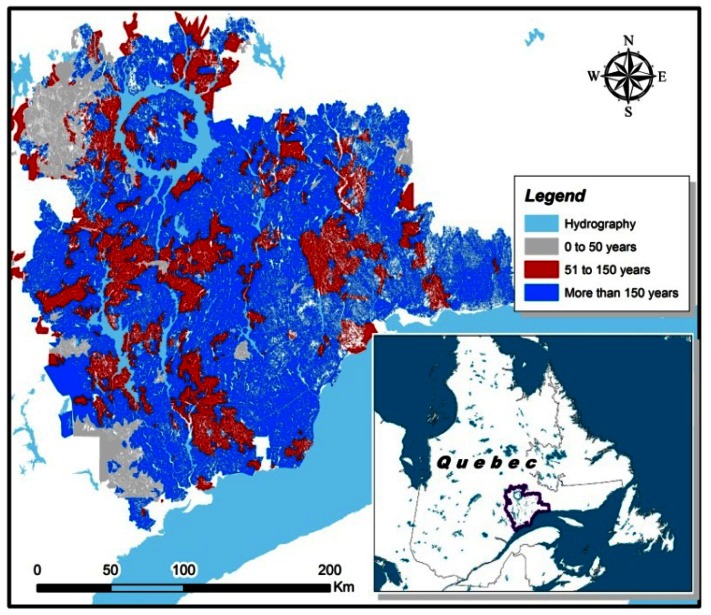
Historical fire map from the North Shore region (data obtained from [23]).

An illustrative example of the methodology used to calculate pass rates for MSR grade 2400Fb-2.0E for the combined TSF classes is shown in Figure 5. For the two higher MSR grades, there was a decrease in pass rates in stands with a TSF greater than 150 years (Table 3). This information is potentially useful to forest planners because it can be used to predict expected MSR lumber recovery based on historical fire maps (Figure 6) overlaid with forest cover. After dividing the North Shore map into just two TSF classes, 50–149 and >150 years, we calculated that in the younger stands, the expected yield of 2100Fb-1.8E and 2400Fb-2.0E lumber grades would be 69% and 43%, respectively, while the corresponding predictions for TSF > 150 years were 58% and 25%. Again, these pass rates should be interpreted with a degree of caution since our predictions assume that the harvest is composed entirely of healthy black spruce trees with no stem decay. Thus, a suitable adjustment must be applied to take into account these and other factors causing any degradation in stem or sawn product quality, such as damage sustained during harvesting operations or sawing defects.

## 4. Conclusions/Outlook

The propagation speed of mechanical waves is fairly well correlated with wood stiffness and, in combination with tree diameter, can be used to make unbiased predictions of tree-level MOE, despite the moderate fit of the initial regression model of static MOE as a function of acoustic velocity and tree diameter (*R^2^* = 0.41). In addition, our results showed that acoustic velocity readings can be affected by the insertion depth of the probes. Therefore, to obtain consistent results, care should be taken to ensure a fixed probe depth, at least for slow-growing black spruce stems with relatively thin sapwood.

Results from our case study confirm that black spruce stands in the North Shore region of Quebec have the potential to produce lumber that meets the requirements for MSR grade 1650Fb-1.5E at a high pass rate, regardless of stand structure. However, we noticed differences between stands of different TSF classes for the MSR grades with higher structural requirements. The results show that the wood from forests with a TSF < 150 years would generally have better mechanical properties than wood from forests with a TSF > 150 years, up to 11% and 18% more for MSR grades 2100Fb-1.8E and 2400Fb-2.0E, respectively. Further work is being conducted to identify the biological causes of this difference. A first hypothesis being tested is that wood properties decline as a result of gradual changes in site and stand characteristics in the absence of fire [42]. Tests will also be performed on clear wood samples to evaluate the possibility that mechanical properties are affected by differences in knot size.

The information obtained from acoustic sensors can help inform stand selection decisions according to the current demand for specific MSR lumber grades. While it will be important to increase our understanding of the effect of TSF in our sampling area, the method presented in this study is applicable to other forest types where different factors may affect wood properties. Standing-tree acoustic sensors such as the ST-300 could be used as part of conventional forest surveys to obtain low-cost assessments of wood properties at the regional scale. For such uses, the strength of the relationship between the acoustic velocity measured on a given tree and the MOE of lumber pieces it produces should not be used as the main indicator of standing-tree tool performance. Instead, future efforts should focus on calibrating this relationship at the population level, *i.e.*, between stand-level MOE estimates obtained using acoustic tools and the grade requirements of the associated structural lumber production.

## Figures and Tables

**Table 1.** Mean and standard deviation of acoustic velocity (tree level) and MOE_est_ (15% MC) for each TSF class (n = 333). Identical letter codes indicates no significant difference in means between TSF classes in Tukey HSD tests (α = 0.05).

**TSF Class (years)**		**Velocity (km·s^−1^)**			**MOE_est_(kN·mm^−2^)**			**DBH (cm)**		
	**n**	**Mean**	**SD**		**Mean**	**SD**		**Mean**	**SD**	
50–99	75	4.48	0.35	B	11.16	1.61	A	15.36	2.73	A
100–149	79	4.66	0.35	A	11.12	1.45	A	19.65	5.16	B
150–199	79	4.40	0.43	B	10.61	1.39	AB	20.53	5.22	B
>200	100	4.42	0.35	B	10.55	1.05	B	20.77	5.26	B

**Table 2.** Parameter estimates (θ_1_ and θ_2_, Equation (3)) of the logistic regression models for the proportion of boards meeting the minimum grade stiffness thresholds (MOE_est_ at 15% MC).

**MSR Grade**	**Intercept**	**MOE_est_**
1650Fb-1.5E	2.2442	-
2100Fb-1.8E	−9.6062	0.9413
2400Fb-2.0E	−31.2750	2.6765

**Table 3.** Percentage of the resource meeting the minimum requirements of certain MSR grades, grouped by TSF (time since last fire) class.

**TSF (years)**	**1650Fb-1.5E**	**2100Fb-1.8E**	**2400Fb-2.0E**
50–99	92.0	67.3	41.8
100–149	92.0	71.1	44.0
150–199	92.0	57.7	28.3
>200	92.0	57.9	20.4

## References

[1] Spelter H., McKeever D., Toth D. (2009). Profile 2009: Softwood Sawmills in the United States and Canada.

[2] Comité Sénatorial Permanent de L'agriculture et des Forêts (2011). Le Secteur Forestier Canadien: Un Avenir Fondé sur L'innovation.

[3] Conférence Régionale des Élus (CRÉ) (2004). Les Réalités de l'Industrie du bois de Sciage sur la Côte-Nord.

[4] (2012). Panorama des Régions du Québec: Édition 2012.

[5] Sathre R., Gustavson L. (2009). Process-based analysis of added value in forest product industries. For. Policy Econ..

[6] (2000). Lumber and Value-Added Wood Products: Special Report.

[7] Garet J., Pothier D., Bouchard M. (2009). Predicting the long-term yield trajectory of black spruce stands using time since fire. For. Ecol. Manag..

[8] Jessome A.P. (2000). Résistance et Propriétés Connexes des Bois Indigènes au Canada.

[9] Reid D.E.B., Young S., Tong Q., Zhang S.Y., Morris D.M. (2009). Lumber grade yield, and value of plantation-grown black spruce from 3 stands in northwestern Ontario. For. Chron..

[10] Zhang S.Y., Chauret G., Ren H.Q., Desjardins R. (2002). Impact of initial spacing on plantation black spruce lumber grade yield, bending properties, and MSR yield. Wood Fiber Sci..

[11] Barnette J.R., Jeronimidis G. (2003). Wood Quality and its Biological Basis.

[12] Murphy G.E., Acuna M.A. (2011). Ranking of four contributions to error in stand-level Douglas-fir log supply and value recovery estimation. Can. J. For. Res..

[13] Wang X., Carter P., Ross R.J., Brashaw B.K. (2007). Acoustic assessment of wood quality of raw forest materials—A path to increased profitability. For. Prod. J..

[14] Carter P., Briggs D., Ross R.J., Wang X. (2005). Acoustic Testing to Enhance Western Forest Values and Meet Customer Wood Quality Needs.

[15] Lasserre J.P., Mason E.G., Watt M.S. (2007). Assessing corewood acoustic velocity and modulus of elasticity with two impact based instruments in 11-year-old trees from a clonal-spacing experiment of *Pinus radiata* D.Don. For. Ecol. Manag..

[16] Auty D., Achim A. (2008). The relationship between standing tree acoustic assessment and timber quality in Scots pine and the pratical implications for assessing timber quality from naturally regenerated stands. Forestry.

[17] Mora C.R., Schimleck L.R., Isik F., Mahon J.M., Clark A., Daniels R.F. (2009). Relationships between acoustic variables and different measures of stiffness in standing *Pinus taeda* trees. Can. J. For. Res..

[18] Wang X., Ross R.J., Carter P. (2007). Acoustic evaluation of wood quality in standing trees—Part I. Acoustic wave behavior. Wood Fiber Sci..

[19] Achim A., Paradis N., Carter P., Hernández R. (2011). Using acoustic sensors to improve the efficiency of the forest value chain in Canada: A case study with laminated veneer lumber. Sensors.

[20] D'Amours S., Ronnqvist M., Weintraub A. (2008). Using operational research for supply chain planning in the forest products industry. INFOR : Information Systems and Operational Research.

[21] Huang C.L. (2005). System and Method for Measuring Stiffness in Standing Trees.

[22] Gao S., Xiping W., Wang L., Allison R.B. Modeling Temperature and Moisture State Effects on Acoustic Velocity in Wood.

[23] Bouchard M., Pothier D., Gauthier S. (2008). Fire return intervals and tree species succession in the north shore region of eastern Québec. Can. J. For. Res..

[24] Garet J., Raulier F., Pothier D., Cumming S.G. (2012). Forest age class structures as indicators of sustainability in boreal forest: Are we measuring them correctly?. Ecol. Indic..

[25] Côté G., Bouchand M., Pothier D., Gauthier S. (2010). Linking stand attributes to cartographic information for ecosystem management purposes in the boreal forest of eastern Québec. For. Chron..

[26] Barrette J., Pothier D., Auty D., Achim A., Duchesne I., Gélinas N. (2012). Lumber recovery and value of dead and sound black spruce trees grown in the North Shore region of Québec. Ann. For. Sci..

[27] Yang K.C., Hazenberg G. (1992). Impact of spacings on sapwood and heartwood thickness in *Picea mariana (Mill.) B.S.P.* and *Picea glauca (Moench.) VOSS*. Wood Fiber Sci..

[28] Grenier Y., Blais L., Lavoie É (1991). Aire minimum d'échantillonnnage ou nombre de points de prisme nécessaires pour établir la structure d'un peuplement inéquienne. Can. J. For. Res..

[29] Thompson I.D., Ortiz A., Jastrebski C., Corbett D. (2006). A comparison of prism plots and modified point-distance sampling to calculate tree stem density and basal area. North. J. Appl. For..

[30] (2011). ASTM D4761-11, Standard Test Methods for Mechanical Properties of Lumber and Wood-Base Structural Material.

[31] (2007). ASTM D1990-07, Standard Practice for Establishing Lumber from In-Grade Tests pf Full-Size Specimens.

[32] (2003). NLGA SPS 2-2003 Special Products Standard for Machine Graded Lumber.

[33] Schielzeth H. (2010). Simple means to improve the interpretability of regression coefficients. Methods Ecol. Evol..

[34] Liu C., Zhang S.Y., Cloutier A., Rycabel T. (2007). Modeling lumber bending stiffness and strength in natural black spruce stands using stand and tree caracteristics. For. Ecol. Manag..

[35] Wang X., Ross R.J., McClellan M., Barbour R.J., Erickson J.R., Forsman J.W., McGinnis G.D. (2001). Nondestructive evaluation of standing trees with a stress wave method. Wood Fiber Sci..

[36] Eckard J.T. (2007). Rapid Screening for Solid Wood Quality Traits in Clones of Loblolly Pine (*Pinus taeda* L.) by Indirect Measuremants. M.Sc. Thesis.

[37] Kettunen P.O. (2006). Wood Structure and Properties.

[38] Koubaa A., Isabel N., Zhang S.Y., Beaulieu J., Bousquet J. (2005). Transition from juvenile to mature wood in black spruce (Picea mariana (MILL. B.S.P.). Wood Fiber Sci..

[39] Zhang H., Wang X., Su J. (2011). Experimental investigation of stress wave propagation in standing trees. Holzforschung.

[40] Lemieux H., Beaudoin M., Zhang S.Y., Grondin F. (2002). Improving structural lumber quality in a sample of *Picea mariana* logs sawn according to the knots. Wood Fiber Sci..

[41] Ruel J.C., Achim A., Espinoza H.R., Cloutier A., Brossier B. (2010). Wood degradation after windthrow in a Northern environment. For. Prod. J..

[42] Barette J., Pothier D., Ward C. (2013). Temporal changes in stem decay and dead and sound wood volumes in the northeastern Canadian boreal forest. Can. J. For. Res..

